# Computer-Aided Diagnosis of Diminutive Colorectal Polyps in Endoscopic Images: Systematic Review and Meta-analysis of Diagnostic Test Accuracy

**DOI:** 10.2196/29682

**Published:** 2021-08-25

**Authors:** Chang Seok Bang, Jae Jun Lee, Gwang Ho Baik

**Affiliations:** 1 Department of Internal Medicine Hallym University College of Medicine Chuncheon Republic of Korea; 2 Institute for Liver and Digestive Diseases Hallym University College of Medicine Chuncheon Republic of Korea; 3 Institute of New Frontier Research Hallym University College of Medicine Chuncheon Republic of Korea; 4 Division of Big Data and Artificial Intelligence Chuncheon Sacred Heart Hospital Chuncheon Republic of Korea; 5 Department of Anesthesiology and Pain Medicine, Hallym University College of Medicine Chuncheon Republic of Korea

**Keywords:** artificial intelligence, deep learning, polyps, colon, colonoscopy, diminutive

## Abstract

**Background:**

Most colorectal polyps are diminutive and benign, especially those in the rectosigmoid colon, and the resection of these polyps is not cost-effective. Advancements in image-enhanced endoscopy have improved the optical prediction of colorectal polyp histology. However, subjective interpretability and inter- and intraobserver variability prohibits widespread implementation. The number of studies on computer-aided diagnosis (CAD) is increasing; however, their small sample sizes limit statistical significance.

**Objective:**

This review aims to evaluate the diagnostic test accuracy of CAD models in predicting the histology of diminutive colorectal polyps by using endoscopic images.

**Methods:**

Core databases were searched for studies that were based on endoscopic imaging, used CAD models for the histologic diagnosis of diminutive colorectal polyps, and presented data on diagnostic performance. A systematic review and diagnostic test accuracy meta-analysis were performed.

**Results:**

Overall, 13 studies were included. The pooled area under the curve, sensitivity, specificity, and diagnostic odds ratio of CAD models for the diagnosis of diminutive colorectal polyps (adenomatous or neoplastic vs nonadenomatous or nonneoplastic) were 0.96 (95% CI 0.93-0.97), 0.93 (95% CI 0.91-0.95), 0.87 (95% CI 0.76-0.93), and 87 (95% CI 38-201), respectively. The meta-regression analysis showed no heterogeneity, and no publication bias was detected. Subgroup analyses showed robust results. The negative predictive value of CAD models for the diagnosis of adenomatous polyps in the rectosigmoid colon was 0.96 (95% CI 0.95-0.97), and this value exceeded the threshold of the *diagnosis and leave* strategy.

**Conclusions:**

CAD models show potential for the optical histological diagnosis of diminutive colorectal polyps via the use of endoscopic images.

**Trial Registration:**

PROSPERO CRD42021232189; https://www.crd.york.ac.uk/prospero/display_record.php?RecordID=232189

## Introduction

Colorectal cancer (CRC) is the third most common cancer based on incidence statistics and the second leading cause of cancer-related deaths worldwide [1]. Most CRCs arise from benign neoplastic polyps, such as adenomas [2]. Colonoscopy with the identification and removal of these neoplastic polyps is a standard screening method for CRC, which has been proven to reduce cancer-related mortality [2,3]. This is because polyp removal through colonoscopy prevents the development of CRCs by interrupting the adenoma-carcinoma sequence, which is the most reliable stepwise pathogenesis of CRC development [4].

With regard to the size of colorectal polyps, 90%-95% of the detected polyps are <1 cm, and about half of them are nonneoplastic [3-6]. In the context of diminutive colorectal polyps (DCPs; ≤5 mm), only 0.5%-1.7% of cases had advanced histology, indicating a lower probability of developing CRCs [4,7-9]. However, current practice points out the removal of all detected polyps and sending them for histologic evaluation [10]. This may help determine the surveillance interval for CRC screening with per-patient risk stratification [8]. However, unnecessary polypectomy carries the risk of procedure-related adverse events and is not cost-effective [4,8].

With the advancement of image-enhanced endoscopy, optical diagnosis has been attempted to predict the histology of the detected polyps during colonoscopy by characterizing the surface morphology. This can reduce the need for histologic evaluation after the removal of neoplastic lesions with a small risk of having an invasive component [11]. The unnecessary removal of benign polyps can be avoided with the adaptation of this technique. Therefore, optical diagnosis using electronic or dye-based methods has been recommended for histological classification in clinical practice [10]. In accordance with this technique, Preservation and Incorporation of Valuable Endoscopic Innovations (PIVI) performance thresholds for in situ endoscopic histology prediction (optical biopsy) required for *resect and discard* and *diagnose and leave* strategies have been suggested for the management of DCPs [12]. For the management of polyps suspected as neoplasm with diminutive size based on the optical biopsy, a *resect and discard* strategy should satisfy >90% agreement in postpolypectomy surveillance intervals compared with histologic assessment [4]. For nonneoplastic polyps <5 mm in the rectosigmoid colon, the negative predictive value (NPV) should be >90% to adopt the *diagnose and leave* strategy based on the optical biopsy in PIVI performance thresholds [4]. However, only studies by experienced endoscopists with a high level of confidence showed benefits in optical biopsy [10]. Subjective interpretability, inter- or intraobserver variability, and the learning curve prohibits the widespread implementation of this technique.

Studies on computer-aided diagnosis (CAD) using deep learning or machine learning methods to define the accuracy of CAD models are increasing [13,14]. The performance of the CAD model was not influenced by the endoscopists’ level of confidence, and the CAD model consistently provided robust answers. However, studies with small sample sizes have inadequate statistical strength. Thus, this study aims to evaluate the diagnostic test accuracy (DTA) of CAD models used for the histologic diagnosis of DCPs using endoscopic images.

## Methods

### Adherence to the Statement of Systematic Review and Protocol Administration

This study was conducted in accordance with the PRISMA (Preferred Reporting Items for Systematic Review and Meta-Analyses) of DTA Studies [15]. The study protocol was registered at the International Prospective Register of Systematic Reviews database before the initiation of the systematic review (CRD42021232189). Approval from the institutional review board of the Chuncheon Sacred Heart Hospital was waived.

### Literature Search

Two authors (CSB and JJL) independently performed a core database search of MEDLINE, PubMed, Embase, and Cochrane Library using common search formulas, from inception to January 2020. Duplicate articles were excluded from the analyses. The titles and abstracts of all identified articles were reviewed, and irrelevant articles were excluded. Full-text reviews were subsequently performed to determine whether the pre-established inclusion criteria were satisfied in the identified literature. References were also reviewed to identify any additional relevant articles. Any disagreements in the results of the search process between the 2 authors were resolved by discussion or consultation with a third author (GHB). The search formulas used to identify the relevant articles are presented in Textbox 1.

**Literature search strategy for the core databases. tiab: searching code for title and abstract; Mesh: Medical Subject Headings; ab,ti,kw: searching code for abstract, title, and keywords; Lang: searching code for language; lim: searching code by limiting certain conditions.**

**MEDLINE (Through PubMed)**

1. *artificial intelligence*[tiab] OR *AI*[tiab] OR *deep learning*[tiab] OR *machine learning*[tiab] OR *computer*[tiab] OR *neural network*[tiab] OR *CNN*[tiab] OR *automatic*[tiab] OR *automated*[tiab]: 502318
2. *diminutive*[tiab] OR *small*[tiab]: 1417047
3. *polyp*[tiab] OR *polyps*[Mesh]: 40395
4. 1 AND 2 AND 3: 128
5. 5-4 AND English[Lang]: 125

**Embase**

1. *artificial intelligence*:ab,ti,kw OR AI:ab,ti,kw OR *deep learning*:ab,ti,kw OR *machine learning*:ab,ti,kw OR *computer*:ab,ti,kw OR *neural network*:ab,ti,kw OR *CNN*:ab,ti,kw OR *automatic*:ab,ti,kw OR *automated*: 638513
2. *diminutive*:ab,ti,kw OR *small*: ab,ti,kw: 2056711
3. *polyp*:ab,ti,kw: 29836
4. 3-1 AND 2 AND 3: 198
5. 4-3 AND ([article]/lim OR [article in press]/lim OR [review]/lim) AND [English]/lim: 104

**Cochrane Library**

1. artificial intelligence:ab,ti,kw or AI:ab,ti,kw or deep learning:ab,ti,kw or machine learning:ab,ti,kw or computer:ab,ti,kw or neural network:ab,ti,kw or CNN:ab,ti,kw or automatic:ab,ti,kw or automated:ab,ti,kw: 56749
2. Mesh descriptor: [polyps] explode all trees: 1087
3. polyp:ab,ti,kw: 2855
4. 2 or 3: 3397
5. diminutive:ab,ti,kw or small:ab,ti,kw: 83388
6. 1 and 4 and 5: 48 trials (2021-1-28)

### Literature Selection Criteria

The literature included in this systematic review should meet the following inclusion criteria: designed to evaluate the diagnostic performance of CAD models in the prediction of histology of DCPs based on endoscopic images; presentation of the diagnostic performance of CAD models, including sensitivity, specificity, likelihood ratios, predictive values, or accuracy, which enabled the estimation of true-positive (TP), false-positive (FP), false-negative (FN), and true-negative (TN) values for the histologic diagnosis of DCPs based on endoscopic images; and studies written in English. The exclusion criteria were as follows: narrative review articles; studies with incomplete data; systematic reviews or meta-analyses; and comments, proceedings, or study protocols. Articles meeting at least one of the exclusion criteria were excluded from this systematic review.

### Methodological Quality Evaluation

Two authors (CSB and JJL) assessed the methodological quality of the final included articles using the second version of the Quality Assessment of Diagnostic Accuracy Studies. This tool comprises four domains: *patient selection*, *index test*, *reference standard*, and *flow and timing*, and the first three domains have an applicability assessment. The 2 authors assessed each part as having a high, low, or unclear risk of bias [16].

### Data Extraction, Primary Outcomes, and Additional Analyses

Two authors (CSB and JJL) independently extracted the data from each included study and cross-checked the extracted data. If the data were unclear, the corresponding author of the study was contacted by email to obtain insight into the original data set. A descriptive synthesis was performed using a systematic review process, and DTA meta-analysis was conducted if the included studies were sufficiently homogenous.

The primary outcomes were the TP, FP, FN, and TN values in each study. For the CAD of the histology of DCPs using endoscopic images, the primary outcomes were defined as follows: TP referred to the number of subjects with a positive finding by a CAD model and have adenomas or neoplasms as evidenced by endoscopic images; FP referred to the number of subjects with a positive finding by a CAD model and do not have adenomas or neoplasms based on endoscopic images; FN referred to the number of subjects with a negative finding by a CAD model and have adenomas or neoplasms as evidenced by endoscopic images; and TN referred to the number of subjects with a negative finding on a CAD model and do not have adenomas or neoplasms based on the endoscopic images. With these definitions, the TP, FP, FN, and TN values were calculated for each included study. If the included studies presented comparative diagnostic performance of endoscopists versus CAD models, the TP, FP, FN, and TN values of endoscopists in each study were also extracted.

For additional analyses, such as subgroup analysis or meta-regression, the authors extracted the following variables from each included study: publication year, geographic origin of the data (ie, Western vs Asian), type of endoscopic images, type of CAD models, location of the DCPs (ie, any colon vs rectosigmoid colon), number of total images included, and type of test data sets (internal test vs external test).

### Statistics

The bivariate method [17] and hierarchical summary receiver operating characteristic (HSROC) method [18] were applied for the DTA meta-analysis. A forest plot of the sensitivity and specificity and a summary receiver operating characteristic (SROC) curve were generated using the bivariate method [17] and HSROC [18] method, respectively. The level of heterogeneity across the included articles was determined by the correlation coefficient between logit-transformed sensitivity and specificity by the bivariate method and the asymmetry parameter *β*, where *β*=0 corresponds to a symmetric receiver operating characteristic curve, in which the diagnostic odds ratio (DOR) does not vary along the curve according to the HSROC method. A positive correlation coefficient and a *β* with a significant probability (*P*<.05) indicated heterogeneity between the studies [18,19]. A visual examination of the SROC curve was also performed to identify heterogeneity. Subgroup analysis by univariate meta-regression using the modifiers identified during the systematic review was also performed to identify the reasons for heterogeneity. The pooled NPV by integrating conditional prevalence with respect to a previous distribution (considering the heterogeneity in prevalence) was calculated using a probability-modifying plot.

STATA software version 15.1, including the METANDI and MIDAS packages, was used for the DTA meta-analysis. The METANDI and MIDAS packages require the inclusion of a minimum of four studies for DTA meta-analysis. Therefore, if less than four studies were included in the subgroup analysis, the Moses-Shapiro-Littenberg method [20], as implemented in Meta-DiSc 1.4 (XI Cochrane Colloquium), was used. Publication bias was evaluated using the Deek funnel plot asymmetry test.

## Results

### Study Selection

**Figure 1:**
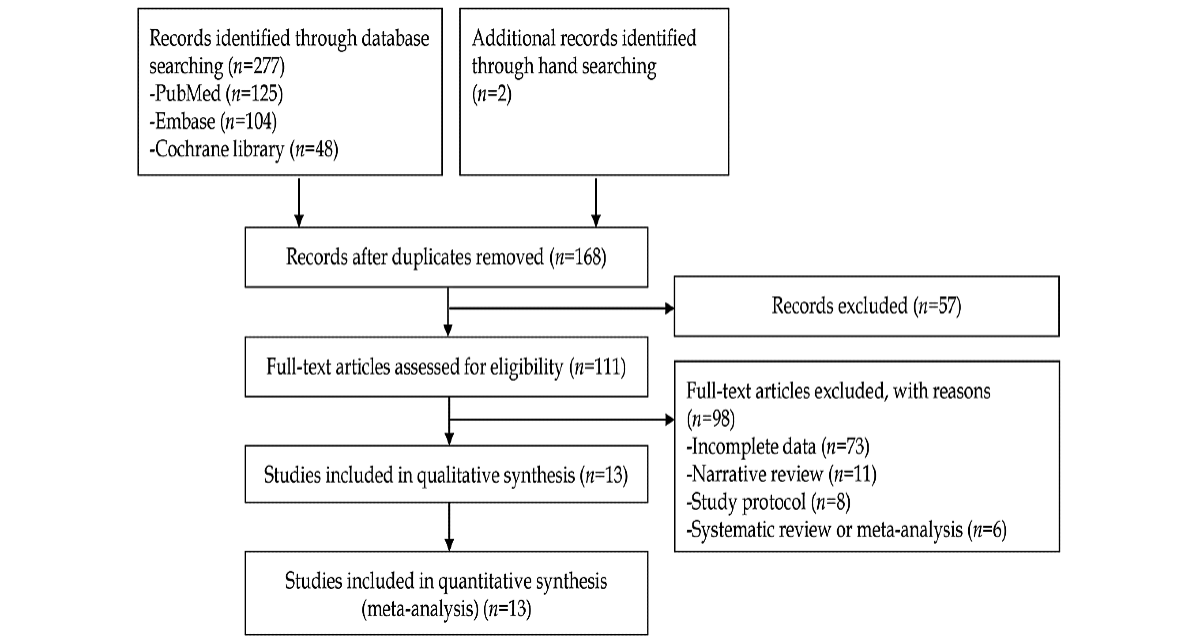
Flowchart of the selection process.

A total of 277 articles were identified following a literature search of the three core databases. Two additional studies were identified by manual screening of the bibliographies. After excluding 111 duplicate studies, 57 additional articles were excluded after reviewing the titles and abstracts. Full-text versions of the remaining 111 articles were obtained and thoroughly reviewed based on the aforementioned inclusion and exclusion criteria. Among these, 98 articles were excluded from the final enrollment for the following reasons: 73 (74%) for incomplete data, 11 (11%) for narrative review, 8 (8%) for study protocol, and 6 (6%) for systematic review or meta-analysis. Finally, 13 studies [21-33] were included in the systematic review. A flowchart of the selection process is presented in Figure 1.

### Clinical Features in Included Studies

The identified studies established and explored the diagnostic performance of CAD models for the classification of adenomatous or neoplastic versus nonadenomatous or nonneoplastic polyps. Among the 13 studies for the CAD of DCPs, 6564 images were identified (3207 cases vs 3357 controls).

Seven studies [24,26,27,29-32] used endoscopic images from Asian populations, and six studies [21-23,25,28,33] used endoscopic images from Western populations. All included studies adopted the definition of DCPs as size <5 mm. However, Shahidi et al [23] adopted a stricter definition for DCPs with a size <3 mm. With regard to the type of CAD model, a deep neural network or convolutional neural network was used in six studies [21-25,30], a support vector machine in six studies [27-29,31-33], and a software-based automatic color intensity analysis in one study [26]. White-light imaging is currently the standard method for inspecting endoscopic lesions. However, one study [28] used white-light imaging to establish a CAD model, and most of the included studies used image-enhanced endoscopic images, such as narrow-band imaging [21-26,30,32,33] or autofluorescence imaging [26] with or without magnification for the detailed characterization of the morphology of DCPs. Three studies [27,29,31] have used images of endocytoscopy, which is a specialized endoscopy that allows the analysis of mucosal structures at the cellular level [34]. With regard to the location of DCPs, most studies [21,23-25,27,28,30-33] did not consider the location of DCPs. However, three studies [22,26,29] separately collected DCPs from the rectosigmoid colon and evaluated the diagnostic performance of the CAD model for these polyps. Most of the included studies [21,23-26,28-33], except for two studies [22,27], have evaluated diagnostic performance using an internal test data set. A study by Zachariah et al [22] presented both external and internal test performance, and a study by Kudo et al [27] presented an external test format of the CAD model previously established by Mori et al in 2016 [31] and 2018 [29,31]. Five studies [25,27,30,31,33] have presented the comparative diagnostic performance of endoscopists versus CAD models for the prediction of histology in DCPs. Detailed clinical features of the included studies are presented in Table 1.

**Table 1:** Clinical characteristics of the included studies.

Study (year)	Nationality (data)	Definition of diminutive polyp	Type of CAD^a^ models	Type of endoscopic images	Type of case and controls	Location of polyps	Type of test data sets	Number of cases in test data set (adenoma)	Number of controls in test data set	TP^b^	FP^c^	FN^d^	TN^e^	Performance of endoscopists (TP/FP/FN/TN)
Eladio Rodriguez-Diaz et al (2020) [21]	United States	≤5 mm	CNN^f^	NBI^g^ with near focus magnification	Neoplastic versus nonneoplastic polyp	All	Internal test	93	75	88	9	5	66	N/A^h^
Zachariah et al (2020) [22]	United States	≤5 mm	CNN	WLI^i^ or NBI	Adenomatous versus nonadenomatous	RS^j^ colon	Internal test	119	472	107	38	12	434	N/A
Zachariah et al (2020) [22]	United States	≤5 mm	CNN	WLI or NBI	Adenomatous versus nonadenomatous	RS colon	External test	183	503	167	60	16	443	N/A
Shahidi et al (2020) [23]	Canada	≤3 mm	CNN	NBI with or without near focus magnification	Adenomatous versus nonadenomatous	All	Internal test	458	186	409	168	49	18	N/A
Jin et al (2020) [24]	South Korea	≤5 mm	CNN	NBI with or without near focus magnification	Adenomatous versus hyperplastic polyp	All	Internal test	180	120	150	10	30	110	N/A
Byrne et al (2019) [25]	Canada	≤5 mm	CNN	NBI with or without near focus magnification	Adenomatous versus hyperplastic polyp	All	Internal test	66	40	65	7	1	33	43/15/9/35
Horiuchi et al (2019) [26]	Japan	≤5 mm	Software-based automatic color intensity analysis	AFI^k^	Neoplastic versus nonneoplastic polyp	All	Internal test	212	217	164	15	48	202	N/A
Horiuchi et al (2019) [26]	Japan	≤5 mm	Software-based automatic color intensity analysis	TME^l^ (WLI, NBI with magnification, and AFI)	Neoplastic versus nonneoplastic polyp	All	Internal test	212	217	191	18	21	199	N/A
Horiuchi et al (2019) [26]	Japan	≤5 mm	Software-based automatic color intensity analysis	AFI	Neoplastic versus nonneoplastic polyp	RS colon	Internal test	65	193	52	9	13	184	N/A
Horiuchi et al (2019) [26]	Japan	≤5 mm	Software-based automatic color intensity analysis	TME (WLI, NBI with magnification, and AFI)	Neoplastic versus nonneoplastic polyp	RS colon	Internal test	65	193	55	8	10	185	N/A
Kudo et al (2019) [27]	Japan	≤5 mm	SVM^m^	Endocytoscope with NBI	Neoplastic versus nonneoplastic polyp	All	External test	1000	680	960	40	40	640	459/12/41/328 (expert); 578/97/422/583 (trainee)
Kudo et al (2019) [27]	Japan	≤5 mm	SVM	Endocytoscope with CE^n^ (methylene blue)	Neoplastic versus nonneoplastic polyp	All	External test	1000	680	960	0	40	680	453/20/47/320 (expert); 690/236/310/444 (trainee)
Cristina Sánchez-Montes et al (2019) [28]	Spain	≤5 mm	SVM	WLI	Neoplastic versus nonneoplastic polyp	All	Internal test	50	50	43	6	7	44	N/A
Mori et al (2018) [29]	Japan	≤5 mm	SVM	Endocytoscope with NBI	Neoplastic versus nonneoplastic polyp	All	Internal test	287	185	268	16	19	159	N/A
Mori et al (2018) [29]	Japan	≤5 mm	SVM	Endocytoscope with CE (methylene blue)	Neoplastic versus nonneoplastic polyp	All	Internal test	287	185	263	17	24	158	N/A
Mori et al (2018) [29]	Japan	≤5 mm	SVM	Endocytoscope with NBI	Neoplastic versus nonneoplastic polyp	RS colon	Internal test	104	144	98	6	6	138	N/A
Mori et al (2018) [29]	Japan	≤5 mm	SVM	Endocytoscope with CE (methylene blue)	Neoplastic versus nonneoplastic polyp	RS colon	Internal test	104	144	96	11	8	133	N/A
Chen et al (2018) [30]	Taiwan	≤5 mm	Deep neural network	NBI with magnification	Neoplastic versus hyperplastic polyp	All	Internal test	188	96	181	21	7	75	367/55/9/137 (expert); 671/95/81/289 (novice)
Mori et al (2016) [31]	Japan	≤5 mm	SVM	Endocytoscope with WLI	Neoplastic versus nonneoplastic polyp	All	Internal test	19	36	18	2	1	34	248/16/25/128 (expert); 646/106/264/374 (nonexpert)
Kominami et al (2016) [32]	Japan	≤5 mm	SVM	NBI with magnification	Neoplastic versus nonneoplastic polyp	All	Internal test	43	45	40	3	3	42	N/A
Gross et al (2011) [33]	Germany	≤5 mm	SVM	NBI with magnification	Adenomatous versus nonneoplastic polyp	All	Internal test	140	135	133	11	7	124	217/17/23/253 (expert); 188/26/52/244 (nonexpert)

^a^CAD: computer-aided diagnosis.

^b^TP: true-positive.

^c^FP: false-positive.

^d^FN: false-negative.

^e^TN: true-negative.

^f^CNN: convolutional neural network.

^g^NBI: narrow-band imaging.

^h^N/A: not applicable.

^i^WLI: white-light imaging.

^j^RS: rectosigmoid.

^k^AFI: autofluorescence imaging.

^l^TME: trimodal imaging endoscopy.

^m^SVM: support vector machine.

^n^CE: chromoendoscopy.

### Quality Assessment of Study Methodology

**Figure 2:**
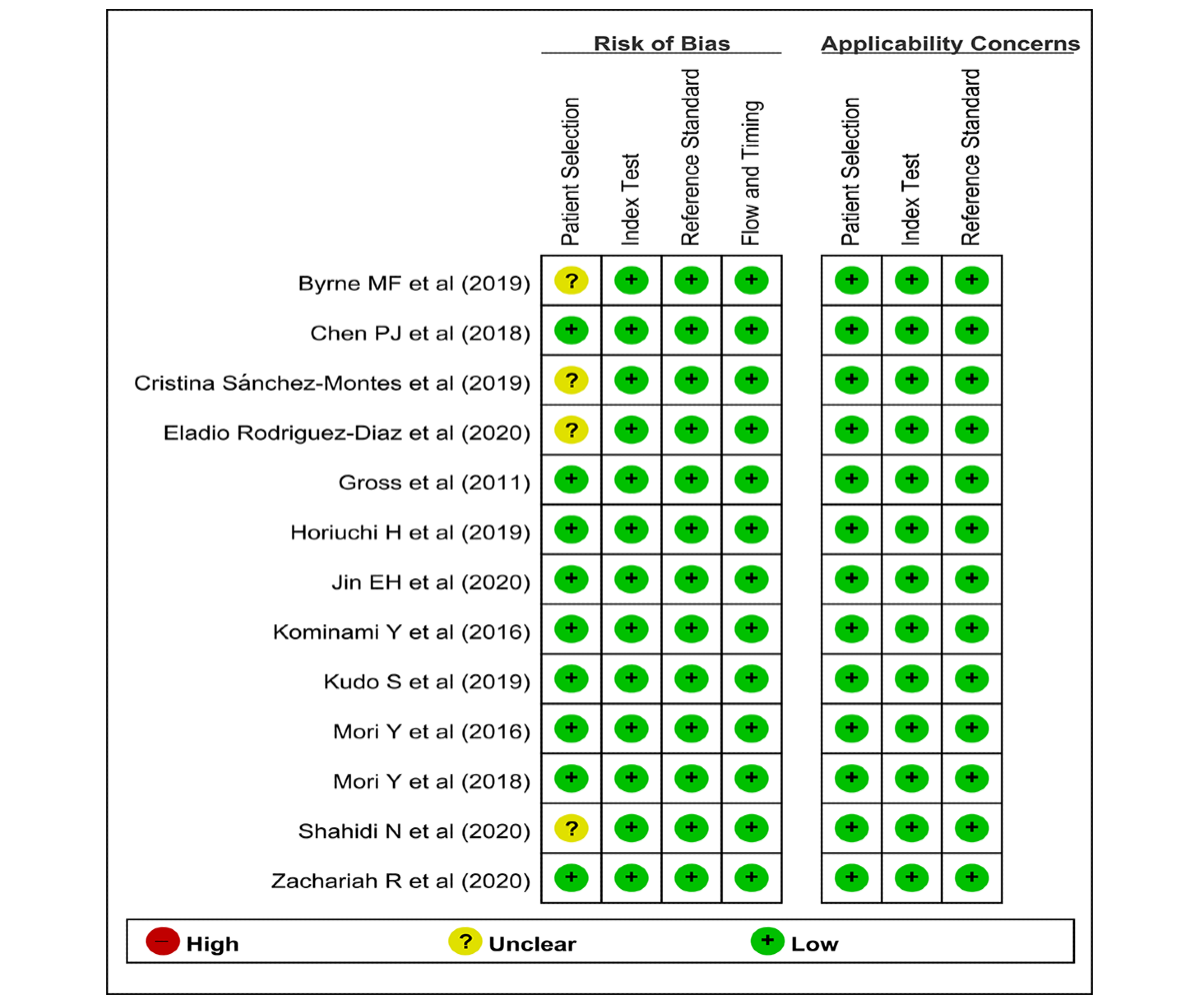
Summary graph of methodological quality. "+" denotes a low risk of bias, "?" denotes an unclear risk of bias, and "−" denotes a high risk of bias.

**Figure 3:**
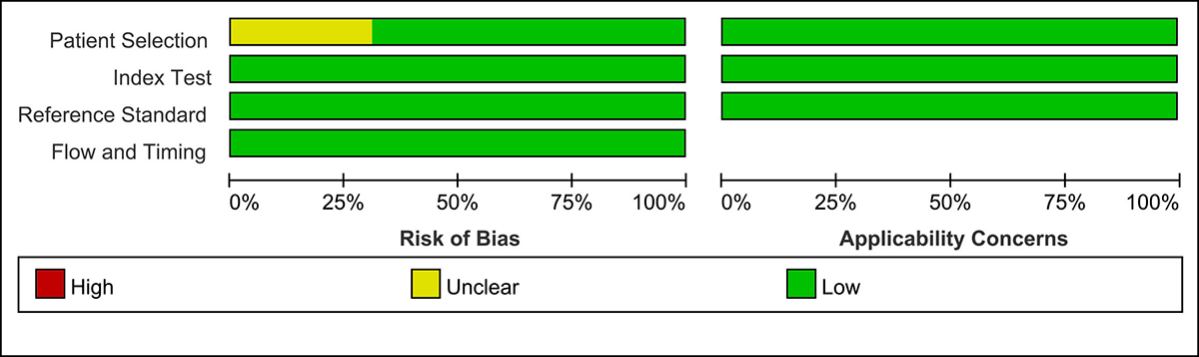
Summary table of methodological quality. "" denotes a low risk of bias, "?" denotes an unclear risk of bias, and "−" denotes a high risk of bias.

The quality of the baseline image data is important because the CAD model is established using the learning features of the baseline training data. Theoretically, the images included in each study should reflect real-world conditions, as the CAD model was established for use in clinical practice. However, as some lesions are rare or abnormal, data imbalance is the main barrier to the learning of CAD models. Most of the included studies in the systematic review attempted to mitigate this pitfall by adopting specific inclusion and exclusion criteria for the enrollment of endoscopic images. However, four studies [21,23,25,28] did not include a detailed description of the image enrollment standard. Therefore, these studies were rated as *unclear risk* in the *patient selection* domain (Figures 2 and 3). This binary classification of *low risk* and *unclear risk* in the *patient selection* domain was adopted as a modifier in the subgroup or meta-regression analysis.

### DTA Meta-analysis of CAD Models

**Figure 4:**
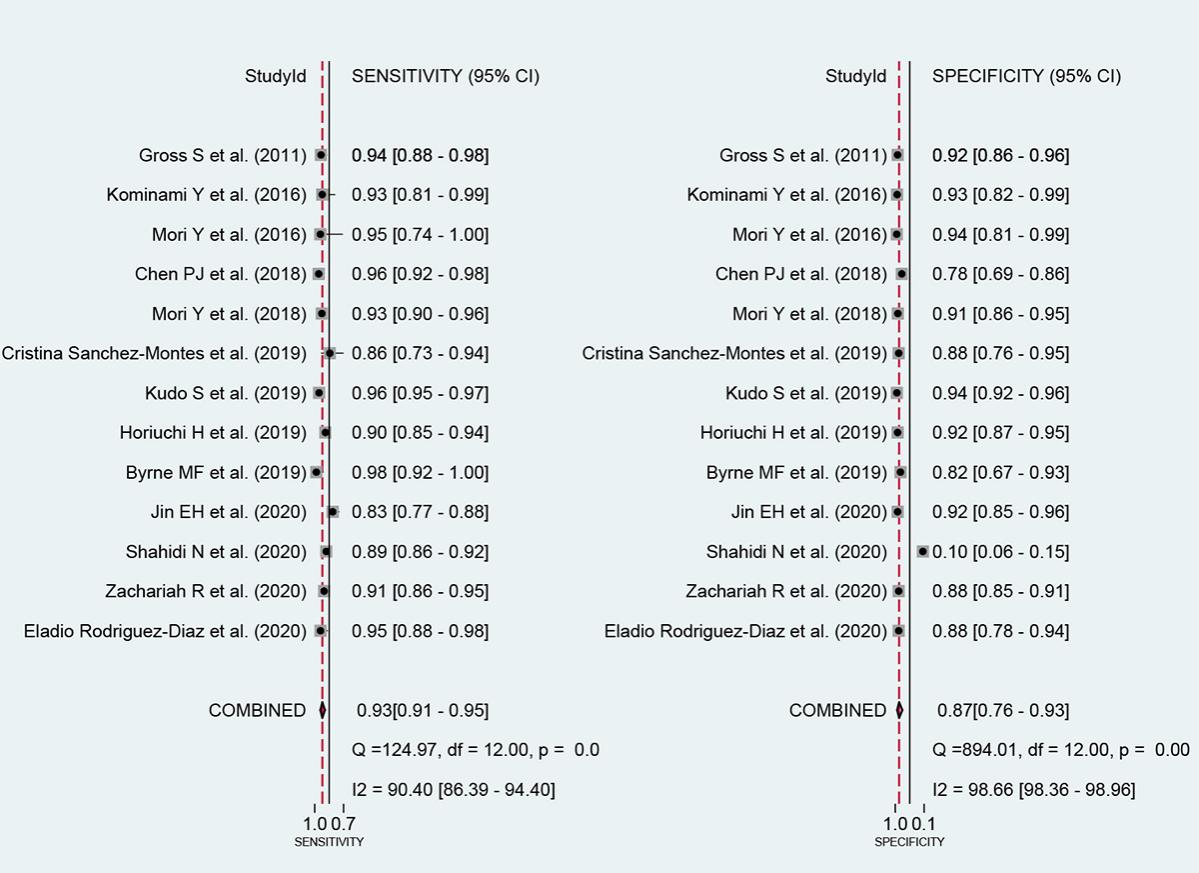
Coupled forest plots of sensitivity and specificity in computer-aided diagnosis models for the diagnosis of histology for diminutive colorectal polyps using endoscopic images.

**Figure 5:**
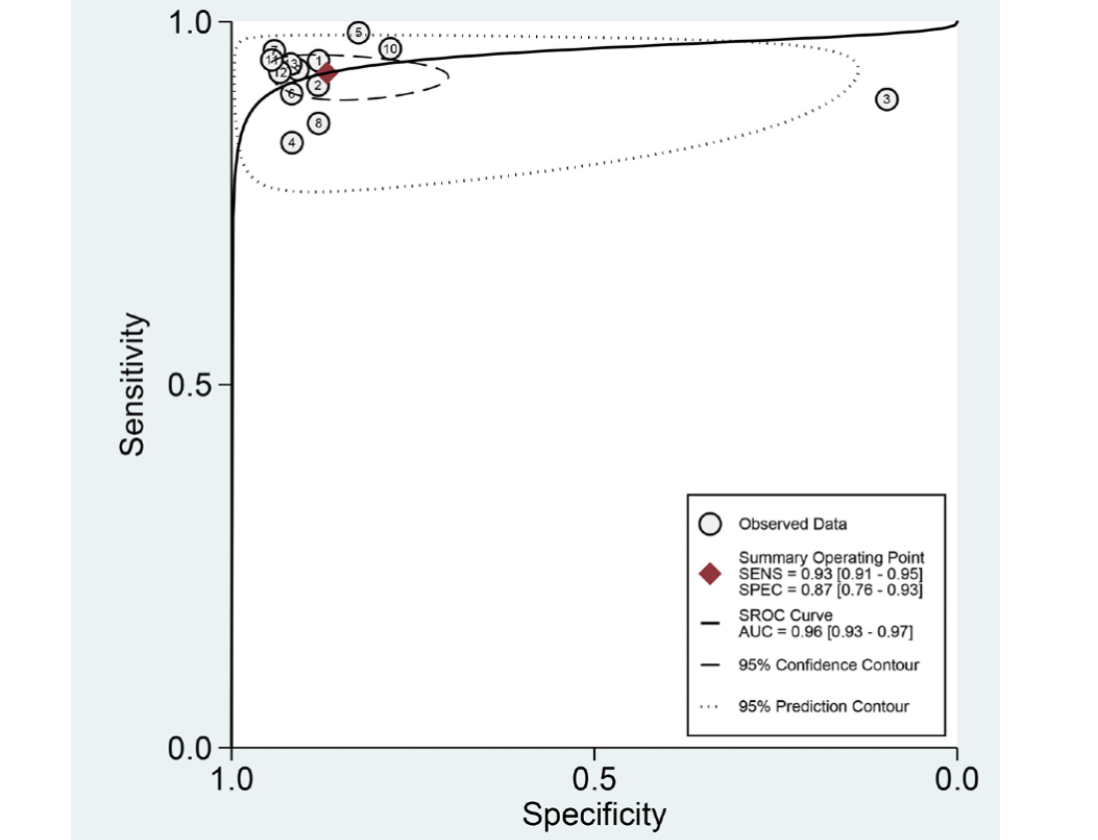
SROC curve with a 95% confidence region and the prediction region of computer-aided diagnosis models for the diagnosis of histology for diminutive colorectal polyps in endoscopic images. AUC: area under the curve; SENS: sensitivity; SPEC: specificity; SROC: summary receiver operating characteristic.

**Figure 6:**
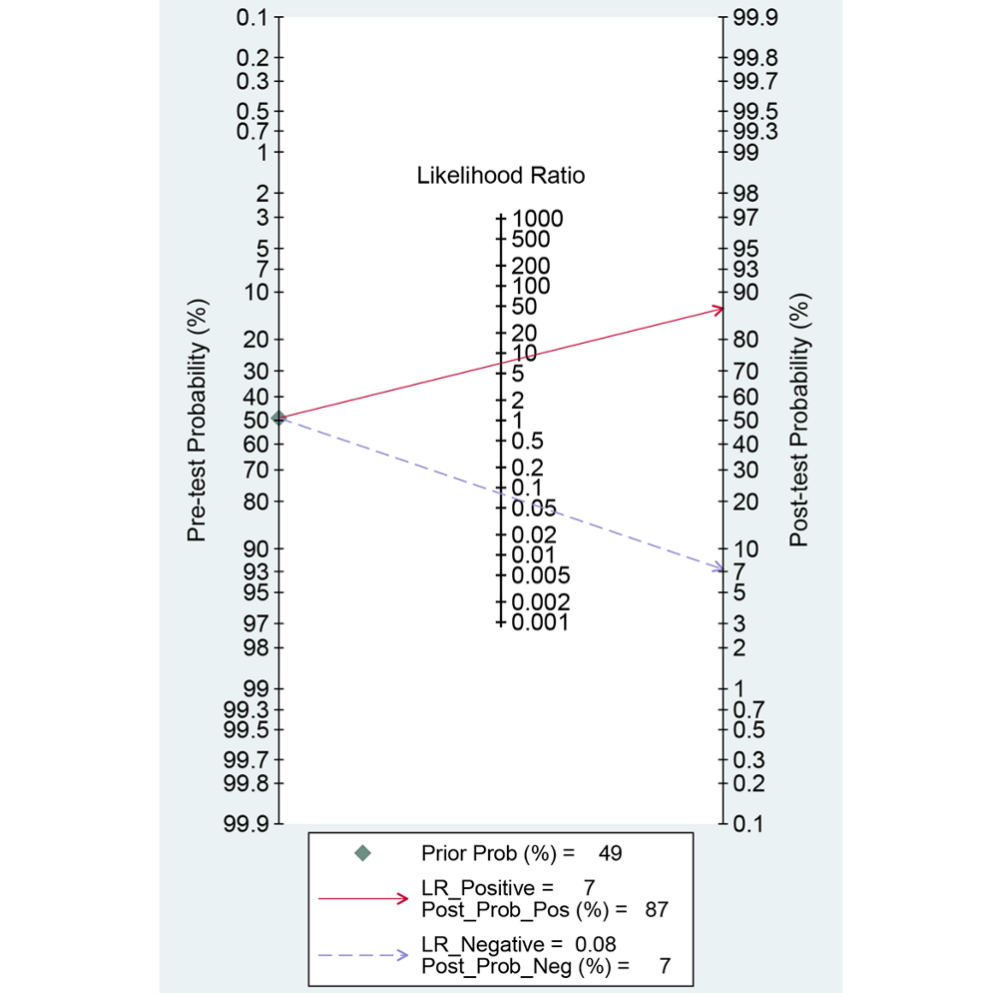
The Fagan nomogram for the diagnosis of histology for diminutive colorectal polyps in endoscopic images. LR: likelihood ratio; Post_Prob_Pos: the posterior probability of adenomas or neoplasms if the finding of the model was positive; Post_Prob_Neg: the posterior probability of adenomas or neoplasms if the finding of the model was negative.

Among the 13 studies [21-33] for the meta-analysis of CAD of DCPs, the area under the curve (AUC), sensitivity, specificity, positive likelihood ratio, negative likelihood ratio, and DOR of CAD models for the diagnosis of DCPs were 0.96 (95% CI 0.93-0.97), 0.93 (95% CI 0.91-0.95), 0.87 (95% CI 0.76-0.93), 7.1 (95% CI 3.8-13.3), 0.08 (95% CI 0.06-0.11), and 87 (95% CI 38-201), respectively (Figure 4; Table 2). The SROC curve is shown in Figure 5. To investigate the clinical utility of the CAD models, Fagan nomogram was generated. Positive findings indicated that adenomas or neoplasms were detected by the CAD models. Negative findings indicated that nonadenomas or nonneoplasms were detected by the CAD models. After assuming a 49% prevalence of adenomas or neoplasms (this value was calculated from the values in Table 1; ie, the total number of cases/controls, ie, 3207/3357, 95.53%), the Fagan nomogram shows that the posterior probability of adenomas or neoplasms was 87% if the finding of the CAD model was positive, and the posterior probability of adenoma was 7% if the finding of the CAD model was negative (Figure 6).

**Table 2:** Summary of diagnostic test accuracy meta-analysis and subgroup analysis for the diagnosis of diminutive colorectal polyps of the included studies.

Subgroup		Included studies (n=13), n (%)	AUC^a^, mean (95% CI)	Sensitivity (95% CI)	Specificity (95% CI)	PLR^b^, mean (95% CI)	NLR^c^, mean (95% CI)	DOR^d^ (95% CI)
Value of meta-analysis in all the included studies		13 (100)	0.96 (0.93-0.97)	0.93 (0.91-0.95)	0.87 (0.76-0.93)	7.1 (3.8-13.3)	0.08 (0.06-0.11)	87 (38-201)
**Comparative performance of CAD^e^ models and endoscopists**								
	Value of CAD models in the comparative analysis	4 (31)	0.96 (0.94-0.98)	0.96 (0.95-0.97)	0.91 (0.84-0.95)	10.5 (5.7-19.1)	0.05 (0.03-0.06)	231 (113-473)
	Value of expert endoscopists in the comparative analysis	4 (31)	0.97 (0.95-0.98)	0.93 (0.89-0.96)	0.89 (0.80-0.95)	8.8 (4.7-16.7)	0.08 (0.05-0.12)	116 (80-168)
	Value of novice endoscopists in the comparative analysis	4 (31)	0.85 (0.82-0.88)	0.78 (0.68-0.86)	0.78 (0.68-0.86)	3.6 (2.3-5.8)	0.28 (0.18-0.43)	13 (6-30)
**Methodological quality of included studies**								
	High	9 (69)	0.97 (0.95-0.98)	0.93 (0.90-0.95)	0.91 (0.88-0.93)	10.2 (7.7-13.5)	0.08 (0.05-0.11)	132 (83-211)
	Low	4 (31)	0.92 (0.89-0.94)	0.93 (0.88-0.96)	0.87 (0.84-0.90)	7.4 (5.9-9.3)	0.08 (0.04-0.14)	96 (52-180)
**Nationality of data**								
	Western	6 (46)	0.93 (0.90-0.95)	0.92 (0.90-0.94)	0.80 (0.51-0.94)	4.6 (1.6-13.7)	0.10 (0.06-0.16)	47 (11-213)
	Asian	7 (54)	0.97 (0.95-0.98)	0.93 (0.89-0.96)	0.91 (0.87-0.94)	10.7 (7.4-15.3)	0.08 (0.05-0.12)	141 (80-248)
**Type of test data sets**								
	Internal test	12 (92)	0.95 (0.93-0.96)	0.92 (0.89-0.94)	0.86 (0.75-0.93)	6.8 (3.5-13.3)	0.09 (0.06-0.13)	76 (32-179)
	External test	2 (15)	Null	0.95 (0.94-0.96)	0.92 (0.90-0.93)	11.1 (4.7-26.4)	0.06 (0.03-0.15)	174 (36-841)
**Location of polyps**								
	All	12 (92)	0.96 (0.93-0.97)	0.93 (0.90-0.95)	0.87 (0.75-0.93)	7.0 (3.6-13.9)	0.08 (0.06-0.11)	89 (36-220)
	Rectosigmoid colon	3 (23)	0.97 (0.94-0.99)	0.91 (0.87-0.94)	0.91 (0.89-0.93)	6.8 (3.5-13.3)	0.09 (0.06-0.13)	76 (32-179)
**Total number of included images**								
	≥200	8 (61)	0.95 (0.92-0.96)	0.92 (0.90-0.95)	0.84 (0.65-0.94)	5.9 (2.4-14.7)	0.09 (0.06-0.13)	66 (20-222)
	<200	5 (38)	0.96 (0.94-0.98)	0.94 (0.90-0.96)	0.89 (0.84-0.93)	7.9 (5.5-11.2)	0.08 (0.04-0.15)	114 (57-230)
	≥300	6 (46)	0.94 (0.91-0.95)	0.91 (0.88-0.94)	0.84 (0.57-0.96)	5.9 (1.8-19.6)	0.10 (0.06-0.17)	57 (12-275)
	<300	7 (54)	0.97 (0.95-0.98)	0.94 (0.92-0.96)	0.88 (0.83-0.92)	8.1 (5.7-11.7)	0.06 (0.04-0.09)	127 (80-203)
**Type of CAD models**								
	Neural network	6 (46)	0.94 (0.92-0.96)	0.93 (0.88-0.96)	0.76 (0.48-0.92)	4.0 (1.5-10.6)	0.09 (0.05-0.18)	42 (10-184)
	SVM^f^	6 (46)	0.97 (0.96-0.98)	0.94 (0.91-0.96)	0.92 (0.90-0.94)	12.1 (9.0-16.5)	0.07 (0.04-0.10)	186 (101-344)
**Type of endoscopic image**								
	Endocytoscope	3 (23)	0.98 (0.94-0.99)	0.95 (0.94-0.97)	0.94 (0.92-0.95)	13.8 (9.8-19.5)	0.05 (0.04-0.08)	248 (109-566)
	Endoscopy	10 (77)	0.95 (0.92-0.96)	0.92 (0.89-0.94)	0.85 (0.70-0.93)	6.0 (2.8-12.7)	0.09 (0.06-0.14)	64 (24-169)

^a^AUC: area under the curve.

^b^PLR: positive likelihood ratio.

^c^NLR: negative likelihood ratio.

^d^DOR: diagnostic odds ratio.

^e^CAD: computer-aided diagnosis.

^f^SVM: support vector machine.

**Figure 7:**
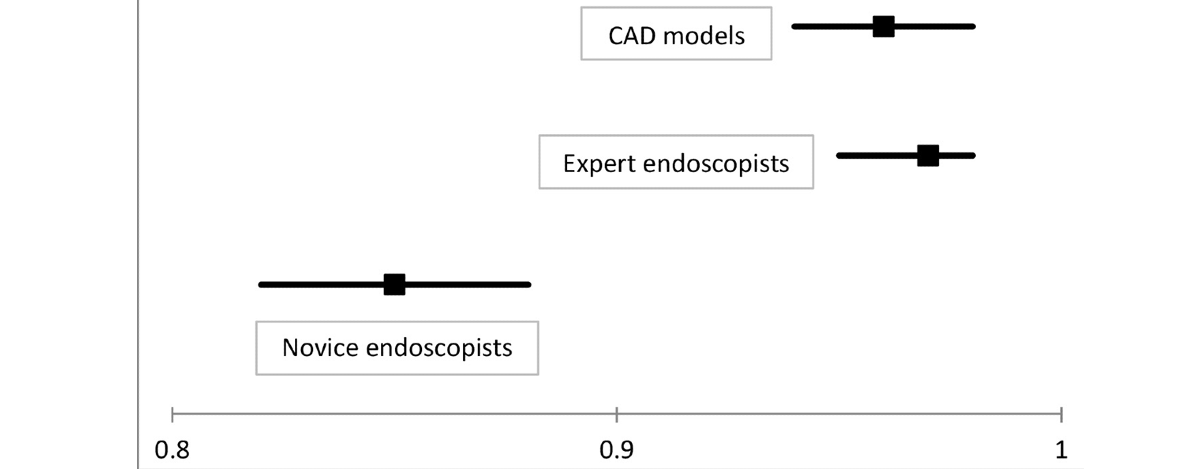
Forest plot of the area under the curve showing the comparative performance between computer-aided diagnosis models and endoscopists for the diagnosis of histology for diminutive colorectal polyps in endoscopic images. CAD: computer-aided diagnosis.

Five studies [25,27,30,31,33] compared the performance of CAD models and endoscopists. Among these, four studies [27,30,31,33] have presented comparative performance between CAD models and endoscopists according to the expertise of the endoscopists (expert endoscopists vs CAD models or novice endoscopists vs CAD models). The pooled AUC, sensitivity, specificity, and DOR of CAD models for the diagnosis of DCPs were 0.96 (95% CI 0.94-0.98), 0.96 (95% CI 0.95-0.97), 0.91 (95% CI 0.84-0.95), and 231 (95% CI 113-473), respectively. For the expert endoscopists, the pooled AUC, sensitivity, specificity, and DOR were 0.97 (95% CI 0.95-0.98), 0.93 (95% CI 0.89-0.96), 0.89 (95% CI 0.80-0.95), and 116 (95% CI 80-168), respectively. For the novice endoscopists, the pooled AUC, sensitivity, specificity, and DOR were 0.85 (95% CI 0.82-0.88), 0.78 (95% CI 0.68-0.86), 0.78 (95% CI 0.68-0.86), and 13 (95% CI 6-30), respectively. The forest plot of AUCs is illustrated in Figure 7, and no significant difference was found between CAD models and expert endoscopists; however, novice endoscopists showed lower pooled AUC for the histologic diagnosis of DCPs than those for CAD models or expert endoscopists.

**Figure 8:**
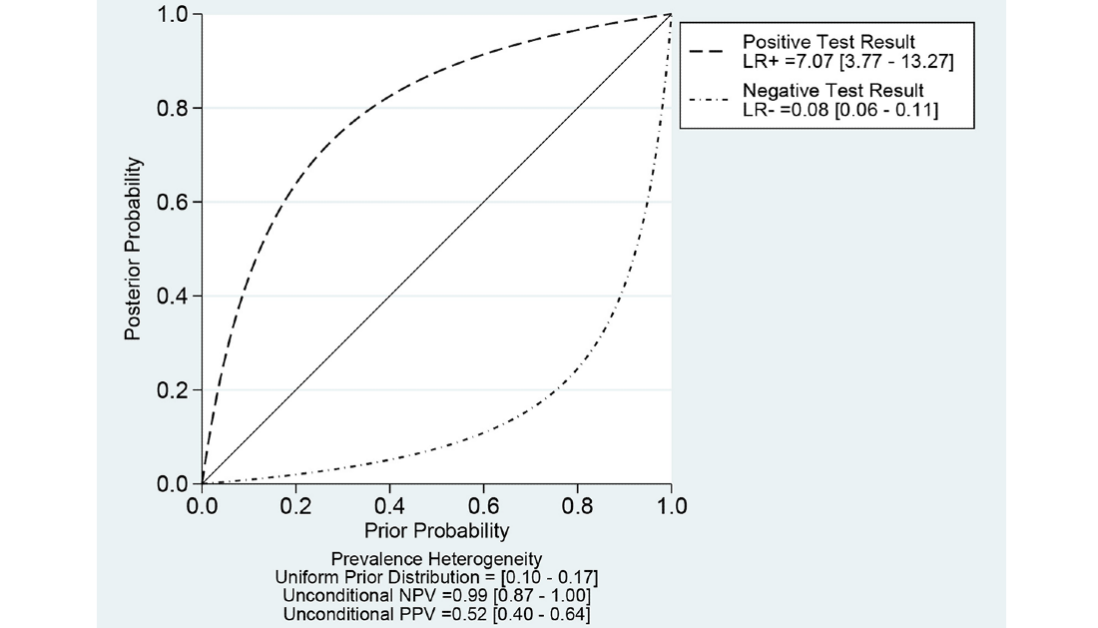
Probability-modifying plot of computer-aided diagnosis models for the diagnosis of adenomatous polyps in the rectosigmoid colon using endoscopic images (assumption of a prevalence of 13.2%). LR: likelihood ratio; NPV: negative predictive value; PPV: positive predictive value.

**Figure 9:**
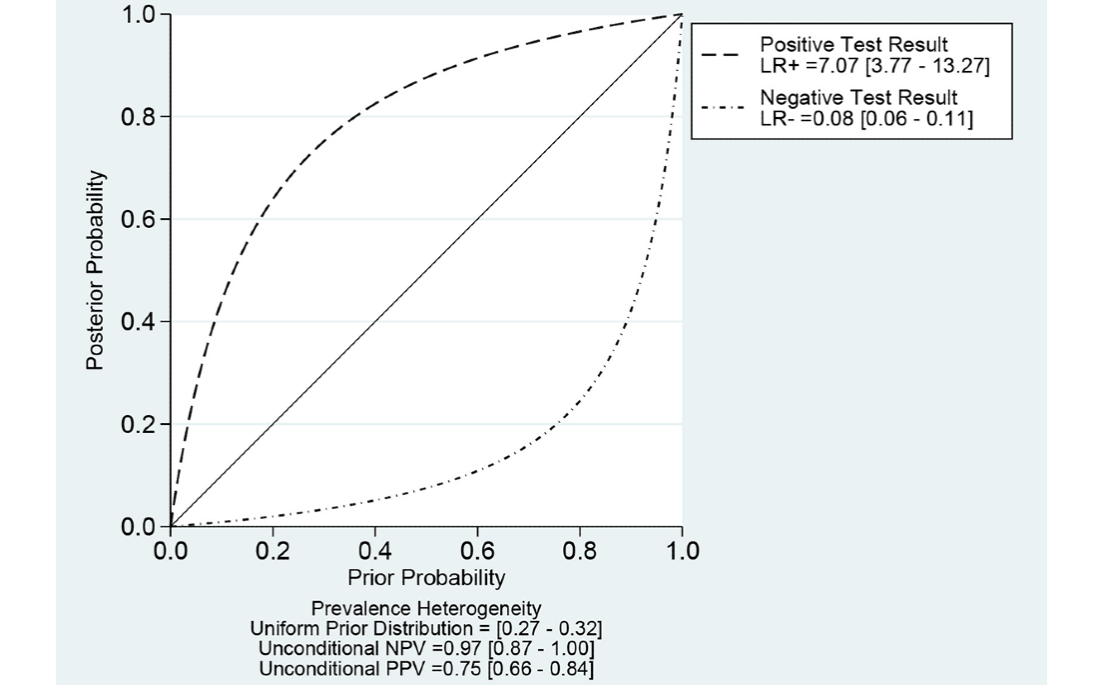
Probability-modifying plot of computer-aided diagnosis models for the diagnosis of adenomatous polyps in the rectosigmoid colon using endoscopic images (assumption of a prevalence of 30%). LR: likelihood ratio; NPV: negative predictive value; PPV: positive predictive value.

With regard to the NPV of CAD models for the diagnosis of adenomatous polyps in the rectosigmoid colon, the pretest prevalence of adenomatous polyp in the rectosigmoid colon was 13.2% (95% CI 10.2%-16.5%) in a recent meta-analysis [35]. For the assumption of this prevalence, the NPV of CAD models was 0.99 (95% CI 0.87-0.99; Figure 8). In this meta-analysis, the prevalence of adenomatous polyp in the rectosigmoid colon was 30% (95% CI 27%-32%) based on 29.53% (352/1192) of polyps in the rectosigmoid colon. For the assumption of this prevalence, the NPV of CAD models was 0.97 (95% CI 0.87-0.99; Figure 9). If we adopt a simple follow-up equation for the NPV of CAD models for the diagnosis of adenomatous polyps in the rectosigmoid colon using pooled sensitivity and specificity, the NPV of CAD models was 0.96 (95% CI 0.95-0.97). The follow-up equation is as follows:

NPV = (specificity × [1−prevalence])/(specificity × [1−prevalence] + prevalence × [1−sensitivity])

### Assessment of Heterogeneity With Meta-Regression and Subgroup Analysis

**Figure 10:**
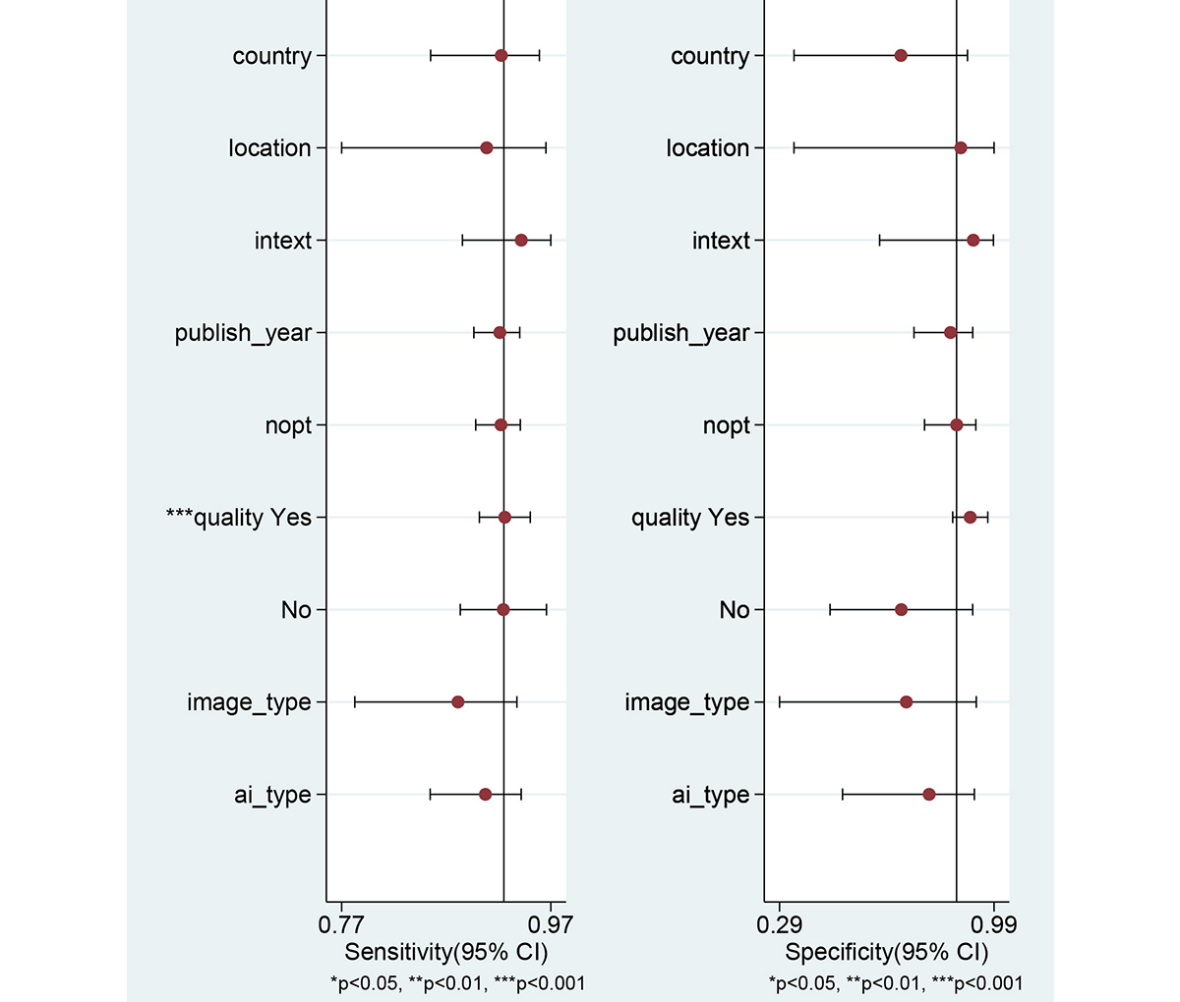
Univariable meta-regression plot of computer-aided diagnosis models for the diagnosis of histology for diminutive colorectal polyps using endoscopic images. ai: artificial intelligence; nopt: number of patients.

First, the authors observed a positive correlation coefficient between the logit-transformed sensitivity and specificity (*r*=0.22) and an asymmetric *β* parameter, with a significant *P* value (*P*=.004), implying that heterogeneity exists among the studies. Second, a coupled forest plot of sensitivity and specificity was obtained (Figure 4). Compared with the enrolled studies, the study by Shahidi et al [23] showed lower specificity. This study was found to have an unclear risk of bias in methodology quality assessment. Therefore, subgroup analysis was carried out according to the methodological quality, and a negative correlation coefficient was found between logit-transformed sensitivity and specificity (*r*=−0.13) and an asymmetric *β* parameter, with a nonsignificant *P* value (*P*=.63) in high-quality studies, indicating an absence of heterogeneity among the studies. Third, the shape of the SROC curve for CAD of DCPs using endoscopic images was symmetric (Figure 5). Fourth, meta-regression using modifiers identified in the systematic review was conducted, and no source of heterogeneity could be identified (published year, *P*=.34; nationality of the data sets, *P*=.29; type of CAD models, *P=*.38; type of endoscopic image, *P=*.23; location of the DCPs, *P*=.90; type of test data sets, *P*=.66; total number of images, *P*=.66; and methodological quality, *P=*.10; Figure 10). Finally, a subgroup analysis based on the potential modifiers was performed, and the pooled AUC of studies with high methodological quality was higher than that of studies with lower methodological quality. Except for this variable (methodological quality), no significant changes in diagnostic performance were found according to the modifiers (Table 2).

### Evaluation of Publication Bias

**Figure 11:**
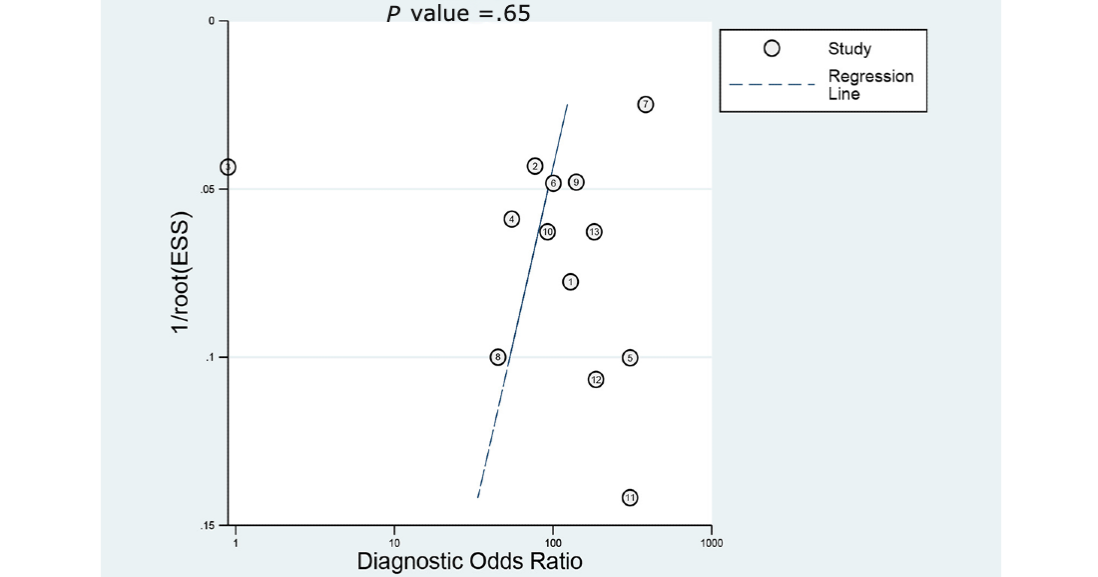
Deek funnel plot of computer-aided diagnosis models for the diagnosis of histology for diminutive colorectal polyps using endoscopic images. ESS: explained sum of squares.

Deek funnel plot of studies for the CAD of DCPs exhibited a symmetrical shape with respect to the regression line (Figure 11), and the asymmetry test showed no evidence of publication bias (*P=*.65).

## Discussion

### Principal Findings

This study presented evidence that CAD models showed high performance values for the histologic diagnosis of DCPs and practical values in the Fagan nomogram, indicating the potential to use these models in clinical practice. This performance was comparable with that of expert endoscopists and higher than that of novice endoscopists. Although the main analysis found heterogeneity among the included studies, the subgroup analysis demonstrated that methodological quality was the reason for the heterogeneity. Thorough meta-regression or subgroup analyses did not reveal any additional reasons for heterogeneity.

Most polyps detected during colonoscopy are diminutive, and considering the low potential of malignancy, resecting all DCPs is not cost-effective [4,7-9]. However, many DCPs are still being resected and sent for histologic evaluation to determine the surveillance interval for CRC screening [10]. CAD models without pathologic diagnosis could lead to cost savings by changing surveillance interval recommendations, and the DTA meta-analysis in our study revealed that the NPV of CAD models for the diagnosis of adenomatous polyps in the rectosigmoid colon was over 90% to adopt the *diagnose and leave* strategy based on the optical biopsy satisfying PIVI performance thresholds for in situ endoscopic histology prediction of DCPs.

Despite the technical challenges of CAD models analyzing a smaller surface area, previous meta-analyses [36-40] have demonstrated that CAD models can increase the adenoma or polyp detection rate, especially for those with small size. With regard to the histologic prediction of DCPs, a previous meta-analysis revealed that endoscopists with only high confidence showed an NPV of approximately 90% using CAD models of digital chromoendoscopy, implicating the potential for adopting the *diagnosis and leave* strategy [41]. Another meta-analysis showed an NPV of 0.95 (95% CI 0.88-0.98) for the CAD of DCPs in nonmagnifying narrow-band imaging [42]. However, the location of the DCPs was not considered, and many studies were omitted from the search process.

An additional finding of this DTA meta-analysis is the robustness of the diagnostic performance of CAD models. The performance values were consistent regardless of the modifiers, except for the methodological quality (Table 2). This was consistent regardless of the nationality of the patients, location of DCPs, total number of included polyps, and type of CAD models or endoscopic images. However, the diagnostic performance of studies with high methodological quality showed higher AUCs than that of studies with low methodological quality, and no evidence of heterogeneity was detected among studies in the subgroup with high methodological quality. Although pooled AUCs in the subgroup of external test datasets could not be measured because only two studies were included in this subgroup, the remaining performance values were comparable with the subgroup of internal test data sets.

### Limitations

Despite the robust evidence in the DTA meta-analysis stated earlier, several inevitable limitations were identified. First, only two or three studies were included in the subgroup analyses of external test data sets, rectosigmoid DCPs, and endocytoscopic images. Bivariate and HSROC methods are advanced statistical techniques that have overcome the limitations of the Moses-Shapiro-Littenberg method (which does not consider any heterogeneity between studies) [43,44]. However, the Moses-Shapiro-Littenberg method is only possible for a subgroup with fewer than four studies. With accumulating evidence on this topic, this statistical pitfall could be overcome. Second, the number of studies was insufficient to enable a comparison of the relative diagnostic performance of endoscopists and CAD models. Considering the real clinical adaptation of endoscopists with CAD models rather than endoscopists versus CAD models, it is no longer necessary to compare the diagnostic capabilities of doctors and CAD models [43]. Owing to the unique characteristics of patients in each institution, CAD models developed from a single institution usually have limitations for widespread implementation, indicating the importance of the external test. However, only two studies conducted external tests to verify CAD model performance. Additional studies focusing on external validation-oriented performance or suggesting a clinical application benefit for future perspectives in established CAD models are expected.

In conclusion, CAD models showed potential for the optical histological diagnosis of DCPs using endoscopic images.

## Abbreviations

AUC: area under the curve
CAD: computer-aided diagnosis
CRC: colorectal cancer
DCP: diminutive colorectal polyp
DOR: diagnostic odds ratio
DTA: diagnostic test accuracy
FN: false-negative
FP: false-positive
HSROC: hierarchical summary receiver operating characteristic
NPV: negative predictive value
PIVI: Preservation and Incorporation of Valuable Endoscopic Innovations
PRISMA: Preferred Reporting Items for Systematic Review and Meta-Analyses
SROC: summary receiver operating characteristic
TN: true-negative
TP: true-positive
